# Diagnostic value of endobronchial and endoscopic ultrasound-guided fine needle aspiration for accessible lung cancer lesions after non-diagnostic conventional techniques: a prospective study

**DOI:** 10.1186/1471-2407-13-130

**Published:** 2013-03-19

**Authors:** Antonio Bugalho, Dalila Ferreira, Ralf Eberhardt, Sara S Dias, Paula A Videira, Felix J Herth, Luis Carreiro

**Affiliations:** 1 Interventional Pulmonology Unit, Hospital Pulido Valente, Lisbon, Portugal; 2 Interventional Pulmonology Unit, Hospital Beatriz Angelo, Av. Carlos Teixeira 3, Loures, 2674-514, Portugal; 3 Chronic Diseases Research Center (CEDOC), Faculdade de Ciências Médicas, Universidade Nova de Lisboa, Lisboa, Portugal; 4 Department of Pneumology and Critical Care Medicine, Thoraxklinik, University of Heidelberg, Heidelberg, Germany; 5 Departamento Universitário de Saúde Pública, Faculdade de Ciências Médicas, Universidade Nova de Lisboa, Lisboa, Portugal

**Keywords:** Lung cancer, Endobronchial ultrasound, Endoscopic ultrasound, Fine needle aspiration, Diagnosis

## Abstract

**Background:**

Lung cancer diagnosis is usually achieved through a set of bronchoscopic techniques or computed tomography guided-transthoracic needle aspiration (CT-TTNA). However these procedures have a variable diagnostic yield and some patients remain without a definite diagnosis despite being submitted to an extensive workup. The aim of this study was to evaluate the efficacy and cost of linear endobronchial (EBUS) and endoscopic ultrasound (EUS) guided fine needle aspiration (FNA), performed with one echoendoscope, for the diagnosis of suspicious lung cancer lesions after failure of conventional procedures.

**Methods:**

One hundred and twenty three patients with an undiagnosed but suspected malignant lung lesion (paratracheal, parabronchial, paraesophageal) or with a peripheral lesion and positron emission tomography positive mediastinal lymph nodes who had undergone at least one diagnostic flexible bronchoscopy or CT-TTNA attempt were submitted to EBUS and EUS-FNA. Patients with endobronchial lesions were excluded.

**Results:**

Of the 123 patients, 88 had a pulmonary nodule/mass and 35 were selected based on mediastinal PET positive lymph nodes. Two patients were excluded because an endobronchial mass was detected at the time of the procedure. The target lesion could be visualized in 121 cases and FNA was performed in 118 cases. A definitive diagnosis was obtained in 106 cases (87.6%). Eighty-eight patients (72.7%) had non-small cell lung cancer, 15 (12.4%) had small cell lung cancer and metastatic disease was found in 3 patients (2.5%). The remaining 15 negative cases were subsequently diagnosed by surgical procedures. Twelve patients (9.9%) had a malignant tumor and in 3 (2.5%) a benign lesion was found. The overall sensitivity, specificity, positive and negative predictive values of EBUS and EUS-FNA to diagnose malignancy were 89.8%, 100%, 100% and 20.0% respectively. The diagnostic accuracy was 90.1% in a population with 97.5% prevalence of cancer. The ultrasonographic approach avoided expensive surgical procedures and significantly reduced costs (p < 0.001).

**Conclusions:**

Linear EBUS and EUS-FNA are able to improve the diagnostic yield of suspicious lung cancer lesions after non-diagnostic conventional techniques. These techniques, performed with one scope, can be offered to patients with accessible lesions as an intermediate step for diagnosis since they may avoid more invasive procedures and hence reduce costs.

## Background

Lung cancer is a major health problem and the most common cause of cancer-related mortality worldwide [1]. In patients with suspected malignant lesions a rapid and precise diagnosis is crucial to determine optimal treatment. Flexible bronchoscopy (FB) and computed tomography-guided transthoracic needle aspiration (CT-TTNA) are the main modalities employed to achieve this purpose. The appropriateness of each method depends on numerous factors such as tumor size and location, accessibility to the primary tumor, local availability of expertise with a particular technique and potential complications of that procedure. The preferable technique is the one that can be performed on an outpatient basis and gives the most information about diagnosis with minimum risk to the patient [2]. CT-TTNA is a standard diagnostic procedure however it cannot be executed in all lesions and holds a significant risk for complications, namely pneumothorax [3]. Flexible bronchoscopy and its ancillary procedures (bronchial washings, brushing and biopsies) are frequently used in lung cancer diagnosis and have a high diagnostic yield in endobronchial tumors but they have a limited ability in diagnosing peripheral, submucosal or peribronchial lesions [4]. The addition of “blind” transbronchial needle aspiration (TBNA) is able to improve the diagnostic yield in some extraluminal tumors but it is influenced by lesion size and location, operator experience, and tumor type, among other factors [5]. Some patients remain without a definitive diagnosis despite being submitted to several procedures and have to undergo a surgical biopsy that, although definitive, is invasive and not always suitable for those with advanced disease and significant comorbidities.

The accuracy and safety of TBNA has been increased by the development of endosonography. Linear real-time endobronchial (EBUS) and esophageal endoscopic ultrasound (EUS) are minimally invasive techniques that are able to image and sample, under direct vision, the intended structures in order to obtain specimens from pulmonary and mediastinal lesions. Both procedures have proven their value, individually or in combination, in mediastinal staging [6,7] but there is limited data on the importance of combining these two techniques in lung cancer diagnosis.

The aim of this study was to evaluate the value of linear EBUS and EUS guided fine needle aspiration (FNA), performed with one echoendoscope, in patients with a suspicious lung lesion who failed diagnosis after conventional procedures.

## Methods

### Patients

Individuals with an undiagnosed suspicious lung cancer lesion who had performed at least one diagnostic attempt with conventional techniques (FB or CT-TTNA) were prospectively enrolled in the study. Patients were eligible if a chest CT documented a tumor arising in an accessible area to EBUS or EUS (paratracheal, parabronchial or paraesophageal lesions) without direct endobronchial signs on a previous bronchoscopy; or if they had a peripheral pulmonary parenchymal lesion (that could not be assessed by linear EBUS and EUS-FNA) with fluorodeoxyglucose (FDG) positron emission tomography (PET) positive mediastinal/hilar lymph nodes (SUV > 3.5). Patients were excluded from the study if the tumor was not abutting the airways or the esophagus; if the primary peripheral lesion was not associated to PET positive mediastinal lymph nodes; or if they already had a confirmed diagnosis of lung cancer and were sent for mediastinal staging.

Based on the described criteria, between June 2008 and June 2011, a total of 123 patients were referred from several institutions for EBUS and EUS-FNA diagnosis. The study was approved by the ethical committee of Centro Hospitalar Lisboa Norte, Hospital Pulido Valente and a written informed consent was obtained in all patients.

### Procedure

All procedures were done in an outpatient setting. EBUS and EUS-FNA were performed under general anesthesia with a flexible ultrasound bronchoscope (BF-UC160F, Olympus, Japan). The scope was first inserted through the tracheobronchial tree towards the area of the target lesion in a standard approach. In the same session, the linear echoendoscope was advanced to the stomach and then slowly withdrawn into the esophagus while making circular movements to localize anatomic landmarks and the intended lesion, according to the previously described technique [7]. The target mass or lymph nodes were identified using ultrasound imaging (EU-C60, Olympus, Japan). Subsequently, the lesions/lymph nodes were punctured at the level of the trachea, main carina, right and left main bronchus, bronchus intermidius, right and left basal trunk and esophagus. A dedicated needle (NA-201SX-4022, Olympus, Japan) was place inside the aimed structures to collect material. At least 4 needle passes were done. A cytophatologist was not present during the procedure and rapid on-site examination (ROSE) was not executed. The operating chest physician judged the macroscopic appearance of each sample and when it was found to be inadequate or insufficient, additional punctures were performed. The aspirated specimens were immediately expelled onto a glass slide, from which a small amount of material was placed on two glass slides and the smears stained using the Papanicolaou technique. Afterwards, a needle wash was made into a container with preservative liquid. The samples were homogenized in a vortex for 10 minutes and centrifuged at 1200 rpm for 5 minutes. The pellet was fixed in PreservCyt solution (Hologic Inc, Iberia), and processed using the T2000 ThinPrep System (Hologic Inc, Iberia). The obtained preparation was stained by the Papanicolaou method for cytological examination. The samples were considered positive (presence of malignant cells), negative (nonexistence of malignant cells, adequate cellular component and absence of bronchial epithelial cells contamination) and inadequate (no cellular component, blood, merely bronchial epithelial cells or insufficient material to achieve a definitive diagnosis). Whenever possible, immunohistochemistry was performed to acquire additional information. A positive result of malignancy was established as evidence and the patient was treated accordingly. Negative and inadequate results were confirmed by subsequent surgical procedures.

### Economic analysis

The estimated costs were based on the Portuguese National Health Service (NHS) according to the Health System Central Administration (ACSS) regulation prices for institutions and integrated services of the NHS (nº 132/2009). Costs were calculated in Euros (€) for patients submitted to conventional techniques and to EBUS/EUS-FNA, and estimated for avoided surgical procedures.

### Statistical methods

The data was entered into a database and analyzed with the SPSS statistical software package (SPSS 18.5 Chicago, Illinois, USA). A descriptive analysis was carried out in which categorical variables were expressed as absolute and relative frequencies and continuous variables as means. Sensitivity, specificity, accuracy, and positive and negative predictive values were calculated using the standard formulas. T-Student tests were used to compare the cost of procedures.

## Results

### Patient characteristics

During the study period, a total of 123 patients met the inclusion criteria (7.9% of all patients submitted to FB or CT-TTNA for lung cancer diagnosis). Of these, 92 were males and 31 females, with a mean age of 63.1 years (range 38–88). Patients’ demographics are summarized in Table 1. One hundred and one patients (82.1%) were current or former smokers with 46.3 packs-year (range 15–125). In 47 cases (38.2%) the pulmonary target lesion was located in the right lung close to central airway, in 28 (22.8%) in the left lung adjacent to the main airways and in 13 cases (10.6%) the mass could only be characterized as central since the radiological findings were confined to hilar and mediastinal structures and did not involve a specific pulmonary lobe. Mean size of lung lesions was 32.1 mm (range 17-64 mm). Thirty-five patients (28.4%) were selected based on the PET-CT scan. In these there was a high primary lung mass FDG-uptake as well as mediastinal and/or hilar PET positive lymph nodes with a mean short-axis diameter of 17.2 mm (range 13-22 mm).

**Table 1:** Patient characteristics

**Patient characteristics**	
Number of patients (n)	123
Mean age (yrs, range)	63.1 (38–88)
Male/female (n, %)	92 (74.8%) / 31 (25.2%)
Location of the target lesion (n, %)	
Left lung mass	28 (22.7%)
Right lung mass	47 (38.2%)
Central pulmonary mass (within hilar and/or mediastinal structures)	13 (10.6%)
Lymph nodes with increase FDG uptake (primary lesion not accessible by linear EBUS or EUS-FNA)	35 (28.5%)
Mean size of the target lesion (mm)	
Pulmonary mass	32.1 mm (17-64 mm)
Lymph nodes short-axis	17.2 mm (13-22 mm)
Non-diagnostic previous investigations (n, %)	
Flexible bronchoscopy	104 (84.5%)
Bronchial/bronchoalveolar lavage	104 (100%)
Bronchial/transbronchial biopsies	91 (87.5%)
Bronchial brushing	59 (56.7%)
“Blind” TBNA	15 (14.4%)
CT-TTNA	19 (15.5%)
≥ 2 non-diagnostic procedures	48 (39.0%)

In 104 cases (84.5%) the patients had been initially submitted to a non-diagnostic FB with accessory sample techniques and in 19 (15.5%) to a CT-guided TTNA. These 19 CT-TTNA cases were all done in peripheral lung lesions. In the subgroup of 13 patients with central lesions, 11 were immediately sent for EBUS and EUS-FNA after the first failed diagnostic attempt (7 cases had been submitted to a blind TBNA). After the initial non-diagnostic exam a second diagnostic procedure has been performed in 48 patients (39.0%) (TTNA in 33 patients and FB in 15 patients) and a third in 10 of these patients (8.1%) (FB in 7 patients and TTNA in 3 patients), before they were sent for EBUS and EUS-FNA. In 64.6% of cases there was a shift between the first and second procedures, this means that patients who have been submitted to FB were afterwards submitted to CT-TTNA and vice versa.

### Procedure details

Mean procedure time was 35.5 minutes (range 21-65 minutes) and all patients were discharged home after the examination. There were no complications related to the procedure. Two patients were excluded from the study since they had direct tumor signs (endobronchial mass) when the endosonography was performed.

In 121 cases the target lesion/lymph nodes could be visualized by EBUS/EUS although FNA could only be performed in 118 cases (97.5%). In three cases there was a major vessel interposition confirmed by Doppler mode that prevented a safe puncture. In 43 patients (36.4%) the target lesion could be assessed by EBUS and EUS-FNA, in 67 patients (56.8%) it could only be punctured by EBUS-TBNA and in 8 cases (6.8%) the lesion was inaccessible through an endobronchial approach and was sampled by EUS. The lesions were punctured 5.4 times (range 4–9 times). A total of sixty-nine lymph nodes were sampled in the 35 patients selected based on the presence of PET positive lymph nodes (1.97 per patient). These were punctured at the level of Mountain-Dressler stations 2R (n = 2), 2L (n = 3), 4R (n = 15), 4L (n = 9), 7 (n = 23), 8 (n = 2), 10R (n = 6), 10L (n = 7) and 11L (n = 2) (Table 2).

**Table 2:** Procedure details

**Procedure details**	
Mean time (range)	35.5 min (21-65 min)
Punctured location	
Left lung mass	
Esophagus	16
Left upper lobe bronchus	8
Left lower lobe bronchus	10
Right lung mass	
Trachea	12
Right main bronchus	9
Right upper lobe bronchus	8
Bronchus intermidius	4
Right middle lobe bronchus	3
Right lower lobe bronchus	8
Esophagus	11
Central pulmonary mass	12
Peripheral lesion with PET + lymph nodes	
Total lymph nodes punctured	69
Right paratracheal superior (2R)	2
Left paratracheal superior (2 L)	3
Right paratracheal inferior (4R)	15
Left paratracheal inferior (4 L)	9
Subcarinal (7)	23
Paraesophageal (8)	2
Right hilar (10R)	6
Left hilar (10 L)	7
Interlobar right (11R)	2
Aspirations per lesion (mean, range)	5.4 (4–9)
Complications	0

EBUS and EUS-FNA provided a definitive diagnosis in 106 cases (87.6%). Of these patients, there were 88 cases (72.7%) of non-small cell lung cancer and the tumor could be characterized as lung adenocarcinoma in 50 patients, squamous cell carcinoma in 23 patients, large cell carcinoma in one patient (14 patients had undifferentiated carcinoma). Small cell lung cancer was identified in 15 patients (12.4%). In three cases the pulmonary lesions were secondary to breast cancer, thyroid adenocarcinoma and renal cell carcinoma. In two cases, malignancy was suspected but could not be confirmed (inadequate sample) and in 10 cases the sample was negative for malignant cells.

**Figure 1:**
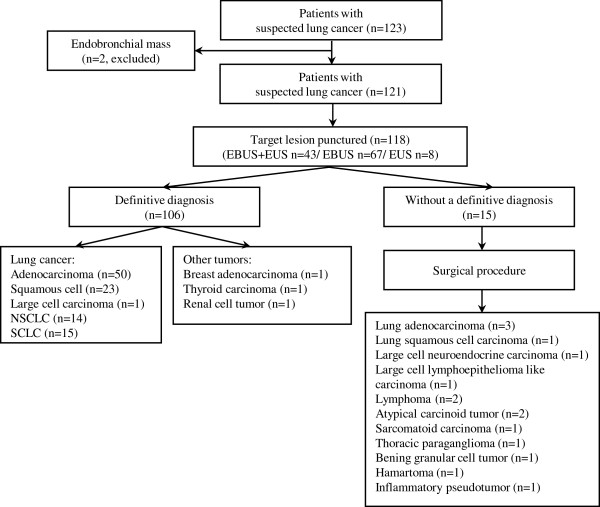
Flowchart of patients enrolled, procedures performed and results obtained.

The fifteen undiagnosed cases were further submitted to mediastinoscopy (n = 2), video-assisted thoracoscopy (n = 5) and open thoracotomy (n = 8). The cytological inadequate samples proved to be lung adenocarcinoma (with extensive necrosis) and large cell neuroendocrine carcinoma on surgery. The negative cases were characterized as atypical carcinoid tumor (n = 2), lymphoma (n = 2), large cell lymphoepithelioma-like carcinoma (n = 1), thoracic paraganglioma (n = 1), sarcomatoid carcinoma (n = 1), hamartoma (n = 1), inflammatory pseudotumor (n = 1) and benign granular cell tumor (n = 1) (Figure 1). Among the cases that the primary lesion could not be punctured one had a final diagnosis of squamous cell carcinoma and two patients of lung adenocarcinoma.

The sensitivity of EBUS and EUS-FNA to diagnose malignancy was 89.8% and specificity was 100%. The positive predictive value and negative predictive value were 100% (95% CI 96.5-100%) and 20.0% (95% CI 7.0-45.2%) respectively. The overall diagnostic accuracy was 90.1% and there were no significant differences between the group of patients with centrally located lesions and PET positive mediastinal lymph nodes. The prevalence of cancer in the study population was 97.5%.

The mean procedure cost for each patient submitted to the non-diagnostic conventional procedures was 273.73€. EBUS and EUS-FNA cost, done with a single scope under general anesthesia, was 740.2€ per patient. The cost of mediastinoscopy or video-assisted thoracoscopy was 2524.25€ per patient and thoracotomy was 5047.22€ per patient. The use of ultrasonography avoided more invasive procedures in 106 cases and led to estimated cost savings of 272382€ (78461€ for EBUS/EUS versus 350843€ for surgical procedures) (p < 0.001). The EBUS and EUS-FNA approach cost saved approximately 2570€ per patient.

## Discussion

The present study demonstrates that in selected patients with central lesions without an endobronchial component, EBUS and EUS-FNA, performed with one linear ultrasound bronchoscope, are accurate methods to diagnose lung cancer.

The diagnosis of indeterminate pulmonary nodules and masses can constitute a considerable challenge in some cases. A broad range of factors must be considered when selecting a specific diagnostic modality to assess a suspicious lesion. These factors include the clinical and radiological information, sensitivity and specificity of the test, invasiveness of the procedure, safety profile, institutional availability of technology, presence of qualified clinicians and cost-effectiveness of the procedure [2].

Several options are available for the diagnosis of indeterminate pulmonary lesions. Less invasive techniques include CT-TTNA and FB however these approaches have both advantages and limitations. FB with its attendant diagnostic modalities is extremely valuable for the diagnosis of a patient with suspected lung cancer but its yield is quite variable. A systematic review [3] reported a diagnostic yield of 88% for centrally located lesions with the combination of different sampling methods (bronchial biopsy, fine needle aspiration, bronchial brushing and washing) therefore in 12% of cases the diagnosis is not reachable by these methods. This percentage is even higher for extraluminal and peripheral lesions since the sensitivity of the FB with all modalities combined is much lower when compared to endobronchial lesions [2,3].

CT-TTNA is frequently used by pulmonologists and interventional radiologists because it provides a more accurate way of diagnosing peripheral pulmonary nodules and masses than FB although it has the potential to cause a non-negligible rate of iatrogenic complications, especially pneumothorax [3]. Its sensitivity clearly depends on the size and location of the lesion, the size and type of the needle, the number of needle passes, and the presence of on-site cytopathology examination [4,8]. The pooled sensitivity of TTNA reported for 12363 patients in 61 studies was 90% [4].

A small yet important number of patients with radiologically suspicious pulmonary lesions remain without a definitive diagnosis after extensive workup (7.9% in our study). Some patients are referred to other procedures such as mediastinoscopy, thoracoscopy or even thoracotomy [9] however they are more invasive, more expensive and associated with increase morbidity and mortality when compared to ultrasonography guided-FNA. Others, not fitted for surgical interventions, are submitted to the repetition of the previous attempted conventional procedures. In the present study, all patients had at least one non-diagnostic FB or CT-TTNA and 39% were submitted to a second non-diagnostic technique which lead to a delay diagnosis and increased patient’s anxiety. Non-ultrasound guided TBNA is a well-established technique for lung cancer diagnosis and mediastinal staging however it is not routinely done in many institutions. In the present study, blind TBNA was attempted in only 14% of cases. The absence of formal training programs, the existence of technical problems with the procedure, concerns regarding its safety, needles cost, inadequate cytopathology support and variable diagnostic yield have been responsible for limited use of this procedure [5,10].

Over the last few years, EBUS and EUS-FNA have been introduced to medical practice and numerous studies have evaluated their impact in lung cancer mediastinal staging.

They have a high diagnostic yield and are simpler, less invasive, cost-effective and allow sampling of lymph node stations not accessible to mediastinoscopy [6]. These techniques have gained their place in lung cancer staging algorithm substituting and complementing more invasive techniques such as mediastinoscopy. Furthermore, real-time linear EBUS and EUS-FNA can constitute an important option to diagnose lung cancer in a single procedure, principally in patients who present centrally located tumors not visible on routine bronchoscopy. Eckardt and coworkers have assessed the value of EBUS-TBNA for the diagnosis of radiologically suspicious chest lesions in a 36 months retrospective study [11]. The technique was able to provide a diagnosis in 55% of cases and the diagnostic yield was higher in central parenchymal lesions compared to enlarged lymph nodes. These results are consistent with the work published by Tournoy et al. [12] that reported a sensitivity of 84% in the diagnosis of central lung lesions not visible at routine bronchoscopy and by Nakajima et al. [13] that obtained 94.1% sensitivity and 94.3% diagnostic accuracy rate in a small population of 35 patients.

EUS-FNA has also been used to diagnose centrally located lung tumors abutting the esophagus [14,15]. The choice between EBUS and EUS totally depends on the availability of equipment, expertise and the location of the suspicious lesion. Two studies [7,16] reported that the combination of EUS-FNA and EBUS-TBNA was better than either alone for mediastinal assessment and were able to achieve a very high diagnostic yield. Herth et al. have shown that the use of a single scope is feasible, effective and safe in primary mediastinal staging [7]. In our population, a final diagnose could merely be achieved by EBUS-TBNA in 56.8% of cases and 6.8% of cases had a definitive diagnosis based of the performance of EUS-FNA. The combination of these procedures accomplished a wider sampling method and there was a clear advantage to perform them in the same setting since it maximized resources. There were also benefits regarding equipment handling and costs by using one ultrasound bronchoscope to perform EBUS and EUS-FNA for diagnostic purposes.

A further relevant point, demonstrated in our study, is that tissue may be acquired from highly suspicious metastatic central lymph nodes to diagnose lung cancer if the main peripheral lesion cannot be easily accessed. Since an important percentage of patients with lung cancer also have mediastinal lymph node enlargement at the time of clinical presentation, EBUS and EUS-FNA can play an important role in the diagnostic algorithm. However, it is important to distinguish patients with an established lung cancer diagnosis sent for mediastinal staging and those referred with enlarged lymph nodes without a definitive diagnosis since the described yield in this second group of patients is usually lower [11,17,18]. In contrast, one of the first published studies reported 96% sensitivity of EUS-FNA in diagnosing mediastinal lymph node malignancy in a small sample of patients with suspected lung cancer in whom bronchoscopy failed to establish the diagnosis [19]. In a more recent study, Lee et al. reviewed their results in a heterogeneous population of 126 patients who underwent EBUS-TBNA to diagnose highly suspicious lung cancer lesions (151 lymph nodes and 44 lung masses) [20]. Eight patients had endobronchial tumor invasion, only 48.4% of patients had a previous attempt to obtain diagnosis and in these cases the most common cytopathological technique was sputum cytology which has a low sensitivity especially when the tumor is extraluminal. The overall sensitivity and diagnostic accuracy was 97.2% and 97.6% respectively. Our results also demonstrate that highly suspicious radiological and PET positive lymph nodes constitute good targets for EBUS and EUS-FNA lung cancer diagnosis. The sensitivity of these minimally invasive techniques combined (89.8%) was lower when compared to their use for mediastinal staging in patients with known lung cancer (96%) [7], however they allowed a broader access to the target lesions compared to other diagnostic studies. In addition, the high lung cancer prevalence in this population – not entirely unexpected since all the included patients had a strong suspicious clinical picture complemented by suggestive accessory exams – and the correct selection of cases that could benefit from these techniques may explain the high sensibility. A further important factor, that may contribute to increase the diagnostic yield in such patients, is related to the number of aspirates per lesion. Lee et al. [21] reported optimal results obtained with successive aspirates until the third EBUS-TBNA pass per lesion and these results were confirmed in subsequent studies [22]. Since the number of needle passes may modify the sensitivity of the technique we have done at least four passes per lesion (mean 5.4/lesion) in order to get an adequate sample.

Another concern regarding the diagnostic procedures is safety. EBUS and EUS-FNA are usually associated with a very good safety profile with a low risk of adverse events. In this study they were also found to be safe since there were no complications associated with the procedures. The use of Doppler mode avoided vessels adjacent or within to the target lesion and in three cases dictated that the lesion could not be safely punctured.

EBUS plus EUS-FNA had a significant influence on the diagnostic management of our patients since they were able to spare more invasive procedures such as surgical exploration in 87.6% of cases. This approach may constitute an important strategy for patients in whom surgery is not an option because of comorbid conditions or advanced metastatic disease.

Regarding the economic impact, a study by Steinfort et al. [23] confirmed that EBUS-TBNA staging of NSCLC was a cost-beneficial strategy in comparison with surgical techniques. For lung cancer diagnosis the use of conventional techniques is still less expensive compared to EBUS and EUS-FNA but our results reinforce the use of linear ultrasonography when there are no endobronchial tumor signs and the intended lesion is adjacent to the main airway or esophagus since it is a cost-effective approach.

Some limitations in our study justify further discussion. It should be noted that it is not a randomized trial and while this is a prospective study, it included a selected population with peripheral and central lung lesions without endobronchial visible signs. Patients were excluded whenever the tumor was not abutting the airways or the esophagus; if there was aerated lung parenchyma surrounding the lesion; or if the primary peripheral lesion was not associated to PET positive enlarged central lymph nodes. Even though the population represented in this study embraces just a part of the suspected lung cancer patients that are frequently assess in the clinical practice we are aware that this particular group of patients usually causes a more challenging diagnosis. Further selection bias may exist since the work was carried out in a referral interventional pulmonology unit although this may be minimized by the fact that in our center CT-TTNA is executed by the authors in association with interventional radiologists. Nevertheless, our data must be confirmed in larger multicenter randomized trials.

Finally, one can always argue that some of the included patients had a primary indication for EBUS or EUS-FNA instead of being submitted to the described conventional procedures. Previous work has shown that EBUS-TBNA can be performed as the first diagnostic procedure in patients with pulmonary masses and concomitant mediastinal lymph nodes [24]. We cannot forget that although EBUS and EUS-FNA are frequently advocated for lung cancer lymph node staging, they are not widely available and, at least in some countries, are performed in selected and referral centers.

## Conclusions

In conclusion, real-time EBUS and EUS-FNA are able to provide a high yield in lung cancer diagnosis after failure of conventional techniques. They may represent an additional step for diagnosing lung cancer since they seem able to replace more invasive diagnostic procedures in selected cases. Both techniques are extremely safe and cost-effective when they are performed by pulmonologists with a single linear ultrasound bronchoscope.

## Abbreviations

CT-TTNA: Computed tomography-guided transthoracic needle; EBUS: Endobronchial ultrasound; EUS: Esophageal endoscopic ultrasound; FB: Flexible bronchoscopy; FDG: Fluorodeoxyglucose; FNA: Fine needle aspiration; NHS: National health system; PET: Positron emission tomography; SUV: Standardized uptake value; TBNA: Transbronchial needle aspiration

## Competing interests

All authors declare that they do not have any competing interests related to this work.

## Authors’ contributions

AB and DF conceived the study, participated in its design, collected the data and drafted the manuscript. RE was involved in the design, progression of the study and helped to draft the manuscript. SSD participated in the design of the study, data collection and statistical analysis. PAV, FJH and LC participated in the coordination of the study and revised the manuscript. All authors read and approved the final manuscript.

## Pre-publication history

The pre-publication history for this paper can be accessed here:

http://www.biomedcentral.com/1471-2407/13/130/prepub
